# Ending TB in South-East Asia: flagship priority and response transformation

**DOI:** 10.1016/j.lansea.2023.100301

**Published:** 2023-10-29

**Authors:** Vineet Bhatia, Suman Rijal, Mukta Sharma, Akramul Islam, Anna Vassall, Anurag Bhargava, Aye Thida, Carmelia Basri, Ikushi Onozaki, Madhukar Pai, Md Kamar Rezwan, Nim Arinaminpathy, Padmapriyadarsini Chandrashekhar, Rohit Sarin, Sandip Mandal, Mario Raviglione

**Affiliations:** a Department of Communicable Diseases, WHO South-East Asia Regional Office, India; b Department of Communicable Diseases, WHO Country Office, Indonesia; c Communicable Disease, BRAC, Bangladesh; d Health Economics, LSHTM, UK; e Department of Medicine, Yenepoya Medical College, India; f USAID, Indonesia; g JATA, Japan; h Department of Epidemiology, Biostatistics and Occupational Health, McGill University, Canada; i Faculty of Medicine, School of Public Health, Imperial College, UK; j National Institute of Research in Tuberculosis, India; k Independent Consultant for Tuberculosis, India; l John Snow Institute, India; m Global Health, University of Milan, Italy

**Keywords:** Tuberculosis, South-East Asia, Flagship

## Abstract

Over the decades, the global tuberculosis (TB) response has evolved from sanatoria-based treatment to DOTS (Directly Observed Therapy Shortcourse) strategy and the more recent End TB Strategy. The WHO South-East Asia Region, which accounted for 45% of new TB patients and 50% of deaths globally in 2021, is pivotal to the global fight against TB. “Accelerate Efforts to End TB” by 2030 was adopted as a South-East Asia Regional Flagship Priority (RFP) in 2017. This article illustrates intensified and transformed approaches to address the disease burden following the adoption of RFP and new challenges that emerged during the COVID-19 pandemic. TB case notifications improved by 25% and treatment success rates improved by 6% between 2016 and 2019 due to interventions ranging from galvanising political commitments to empowering and engaging communities. Cumulative TB programme budget allocations in 2022 reached US$ 1.4 billion, about two and a half times the budget in 2016. An ambitious Regional Strategic Plan towards ending TB, 2021–2025, identifies priority interventions that will need investments of up to US$ 3 billion a year to fully implement them. Moving forward, countries in the Region need to leverage RFP and take up intensified, people-centred, holistic interventions for prevention, diagnosis, treatment and care of TB with commensurate investments and cross-ministerial and multi-sectoral coordination.

## Introduction

Tuberculosis is one of the oldest health problems afflicting humanity. Despite being a preventable and curable disease, it has remained one of the leading killers among infectious diseases for decades.

In the past three decades, the world has seen renewed efforts to address TB with WHO and partners galvanizing the international community into action. Given the relationship between social development and a healthy workforce, control of infectious diseases was included in the Millennium Development Goals (MDG) framework. Significant gains were made to achieve the MDG target of stopping the increase in incidence and decreasing the mortality of TB between 1990 and 2015. This success encouraged the global community to set more ambitious goals—ending the priority diseases including TB by 2030 under the Sustainable Development Goals (SDG) framework. From TB control in the MDG era to ending TB in the SDG era marked a paradigm shift.

The SDG-3 focused on “ensuring healthy lives and promoting well-being for all at all ages” and target 3.3 calls for the elimination of high-burden communicable diseases—specifically TB, HIV/AIDS, malaria and neglected tropical diseases. The adoption of SDGs coincided with the historic resolution adopted in the 67th World Health Assembly to end TB by 2035. The strategy includes incidence and mortality milestones to be achieved by 2020 and 2025. Importantly, the strategy also focused on social aspects with the milestone for 2020 that no TB patients and their households face catastrophic costs because of the disease.

The funding support from the Global Fund to Fight AIDS, Tuberculosis, and Malaria (the Global Fund or GF) and bilateral donors like USAID facilitated the work of national programmes and technical partners to implement ambitious strategies to end TB worldwide.

The global goal of ending TB depends on the progress made in the WHO South-East Asia (SEA) Region which accounts for 45% of the global incidence of TB and more than half of its global mortality. Patient cost surveys in countries of the Region have revealed that 30%–80% of households incur catastrophic costs due to the disease forcing families to sell their assets and pull children out of school.

Given the regional burden and the need to gather momentum to end TB, the Regional Director of the WHO SEA Region in 2017 declared ‘Accelerate Efforts to End TB by 2030’ a Regional Flagship Priority (RFP) Area. By Since then, several transformative and evidence-based changes to end TB have been implemented in the Region. This article aims to illustrate these approaches and highlight new challenges emerging due to the COVID-19 pandemic. The article presents the way forward, emphasising the need for the continuity of TB as a flagship priority to achieve the 2030 SDGs.

## TB disease burden in the WHO SEA Region

In 2021, the estimated incidence rate of TB in the WHO SEA Region was 234 per 100,000 population, which means that 4.8 million new cases emerged. About 38% of this number of estimated patients was either not diagnosed or not reported in the health system. The HIV-negative TB mortality rate for the Region was 37 per 100,000 population translating into 763,000 deaths or more than half of global TB deaths in 2021. Another 25,000 deaths occurred in the same year in the Region among people living with HIV (Table 1).^1^ TB was also the third highest cause of disability-adjusted life years (DALYs) lost in the Region (2019) among the most productive age group (15–49 years),^2^ reflecting the impact of TB on social and economic development.

**Table 1:** Incident TB case and MDR/RR-TB case notification gap in SEA Region (2021).

	TB			MDR/RR-TB		
	Estimated new/relapse number	Notified	Gap	Estimated annual number	Notified	Gap
Bangladesh	375,000	306,701	18%	4500	1601	64%
Bhutan	1300	854	34%	180	58	68%
DPRK	133,000	87,413	34%	5300	270	65%
India^a^	2,950,000	1,965,444	33%	119,000	58,837	51%
Indonesia	969,000	432,577	55%	28,000	8268	70%
Maldives	200	87	57%	2	0	NA^b^
Myanmar	194,000	64,410	67%	9700	1679	83%
Nepal	69,000	28,252	59%	2800	687	75%
Sri Lanka	14,000	6551	53%	91	10	89%
Thailand	103,000	71,488	31%	2400	844	65%
Timor-Leste	6400	3193	50%	57	20	65%
SEA Region	4,814,900	2,966,970	38%	172,030	72,274	58%

a Interim estimates of incidence.

b Very small number to be represented in percentages.

In 2021, the Region also contributed to 38% of the global MDR/RR-TB (rifampicin-resistant) burden. The Region notified only 42% of the estimated MDR-TB incidence of the Region in 2021.

Surveys on catastrophic costs due to TB for families conducted in 4 countries in the Region—Indonesia, Myanmar Thailand and Timor-Leste show that 30%–80% of TB-affected families face catastrophic costs.^3^, ^4^, ^5^, ^6^ This pushes already fragile communities and families into further impoverishment. Some other smaller studies in other countries of the Region yielded similar results (Table 2).

**Table 2:** Estimates of the financial implication of TB on patients and their households in Countries of WHO-SEA Region.

Country (Source)	Key methods	Financial impact of TB
Bangladesh^7^	Data on income, direct and indirect patient costs, asset sales, and loans were collected in 2011/2012 for 96 patients in Bangladesh	53% dissaving rate (includes taking of loans or selling assets. Used as a proxy for catastrophic costs by authors)
India^8^	A cross-sectional study of 455 individuals with TB	31% of households face catastrophic costs
Indonesia^4^	Analysis of financial implications for 282 TB and 64 MDR-TB patients	36% of households face catastrophic costs–(43% among poor households)
Myanmar^6^	National Patient Cost Survey in 2015	48% of patients faced income loss
Nepal^9^	Ninety-nine patients interviewed (50 active case finding and 49 passive case finding)	Reduction in household income of 37% and 38% for active case finding and passive case finding patients, respectively
Thailand^5^	National Patient Cost Survey in 2020	30% of households face catastrophic costs
Timor-Leste^3^	National Patient Cost Survey in 2017	80% of households face catastrophic costs

Six of the 11 countries in the SEA Region – Bangladesh, DPR Korea, India, Indonesia, Myanmar and Thailand – are among the high-burden countries (HBC) while for the high MDR-TB burden countries, Nepal replaces Thailand in the list. Although not listed among HBCs, Timor-Leste is among the top 10 high-incidence rate countries globally.^1^

## Transformative approaches embraced following the adoption of TB as a flagship priority

Declaration of TB as a flagship priority has helped garner an exponential increase in political commitment through a sustained engagement at the highest political level, including the heads of State and health ministers. The Region has adopted a 360-degree approach with engagement of all stakeholders in the fight against TB. This followed the pioneering work in evidence-based modelling to identify priority interventions undertaken by the WHO SEA Regional Office, in close collaboration with partners and various technical experts, as detailed below.

### Intensified political commitment

The Delhi Call for Action to End TB, 2017^10^ brought together health ministers to garner commitment to “bend the curve”. This meeting built momentum towards the global ministerial meeting in Moscow held in November 2017.^11^ Subsequently, the Delhi End TB Summit held in March 2018^12^ reviewed progress since the adoption of the Delhi Call for Action a year ago. The 2018 UN General Assembly High-Level Meeting on TB^13^ committed to greater efforts and investments towards increased coverage for realising the goal of ending TB. The next SEA Region high-level ministerial meeting for renewed TB response in 2021 was co-hosted by India, Indonesia, and Nepal^14^ led to a commitment for a renewed response to ending TB during the COVID-19 era. More recently ‘Gandhinagar Declaration’ was adopted by countries of the SEA Region during the high-level meeting in August 2023 that calls for establishment of multisectoral platforms in countries, improved access to TB services through equitable, rights-based approach and allocation of needed resources for improved coverage and addressing social determinants of TB. Several country-level initiatives have also been undertaken to demonstrate the translation of political commitment to realities (Box 1).

Box 1 **Political commitment translating into action in countries.**

**Table:** 

India	
Commitment	Impact
In 2018, India's Prime Minister shared a bold vision of ending TB in the country by 2025. Among many new initiatives, an innovative approach to involve communities in patient support–“*Pradhan Mantri TB Mukt Bharat Abhiyan*” (Prime Minister's TB Free India Campaign)–was launched in 2022 by the President of India.^15^ Under this programme, TB patients can be cared for by an individual, elected representatives or institutions by enrolling themselves as *Ni-kshay Mitra* to provide nutritional and treatment support to adopted patients.	As per the information available on the *Ni-kshay* website, there are close to 90, 000 *Ni-kshay mitras* registered providing support to more than 1 million TB patients^16^

**Indonesia**	
**Commitment**	**Impact**
In 2021, Indonesia issued a Presidential Decree to End TB that mandates an aggressive tracking of TB cases, availability of TB drugs and prevention efforts to reach the end TB goal of 2030.^17^	Following the President's decree, WHO worked with the Ministry of Health and the Coordinating Ministry for Human Development and Culture to establish a National Partnership Network for TB Control which is accountable to the President. The forum consists of partners from ministries, NGOs, patient networks, universities as well as development partners It is an important step towards realizing the Multisectoral Accountability Framework (MAF-TB).

**Maldives**	
**Commitment**	**Impact**
The country with its low TB incidence rate of around 30/100,000, aims to end TB by 2025. Several partners have come together to support the country in its endeavour by providing technical and strategic assistance, notably Aurum Institute, the Indian Council of Medical Research (ICMR) and the US CDC, facilitated by WHO.	The country has received the support for 1000 patient courses of Rifapentine and is in negotiation with ICMR to receive a grant of over US$ 1.5 million under the Regional Research Platform.

**Myanmar**	
**Commitment**	**Impact**
TB funding and government spending on TB control increased annually following the declaration of TB as a Regional Director's Flagship priority. The expansion of TB care in public–private mix (PPM) model that engages both public and private providers has played an important role by increasing case finding and quality of care. A National Strategic Plan (NSP) was developed in 2010.^5^	Myanmar, a high TB burden country, is the only one in the Region that has achieved the 2020 milestone for the End TB target of a 20% reduction in TB incidence rate and a 35% reduction in TB deaths from the 2015 baseline.

**Nepal**	
**Commitment**	**Impact**
The country launched its “TB-Free Nepal Declaration” initiative in 2021, which is a ministerial commitment to end TB and accelerate interventions at the local level.^18^ It aims to intensify TB case findings, expand access to preventive treatment, and enhance quality treatment, care and social schemes.	The initiative is a renewed comprehensive approach to strengthen the ownership and accountability of local-level governance in TB control and management. An improved case notification and treatment outcome has been observed in several *nagar pallikas* implementing the initiative

**Timor-Leste**	
**Commitment**	**Impact**
In 2021, Timor-Leste's Ministry of Health held a multi-stakeholder pledge signing ceremony with support from the WHO Country Office.^19^ The pledge envisions comprehensive support and action to end the TB epidemic in Timor-Leste. At the same time, the National Plan for Accelerated Actions for Ending TB by 2025 was launched.	The country has embarked on a digital technology-supported Vulnerability Assessment that identifies high-risk groups in the study population. The approach is planned to be expanded to the entire country for screening and early identification of TB.

### Strengthened technical and strategic support

**Fig. 1:**
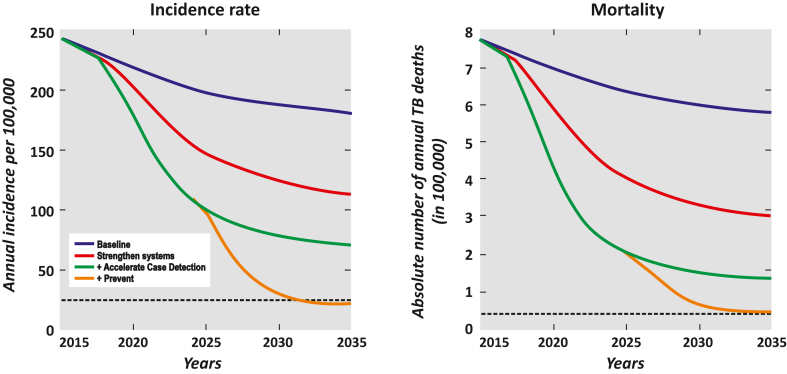
**Impact of three packages of interventions on TB incidence and mortality in SEA Region**.

Source: Ending TB: Invest Now or Pay Later. New Delhi: World Health Organization, Regional Office for South-East Asia; 2018.^22^

The elements for ending TB were published in WHO report “Implementing the End TB Strategy: The Essentials”^20^ and the Global Plan to End TB of Stop TB Partnership.^21^ The SEA Region generated region-specific information to contextualise such global guidance. A modelling study^22^ commissioned by WHO in 2018 demonstrated that none of the Member States of the Region would be able to meet the 2030 target of ending TB at the with the prevailing state of action. The study guided the need for intensified strategies such as greater focus on prevention and the need for additional investments (Fig. 1). The document highlighted funding needs in the Region for aspirational targets of coverage required for ending TB. These findings acted as a catalyst to bring the Member States together and adopt the Delhi Call for Action in 2017.

The Region also came up with evidence on optimizing active case-finding for TB.^23^ During the COVID-19 pandemic, virtual and hybrid modalities were used to spread awareness around the updated WHO guidelines for TB diagnosis and management. A virtual workshop on strengthening the capacity of laboratory staff for second-line drug-susceptibility testing and genome sequencing was held in collaboration with Vita-Salute San Raffaele University, Milan (WHO Supranational Reference Laboratory), in August 2021.

### Bolstered community empowerment and engagement

Community engagement in health care planning, delivery and monitoring, has long been identified as a priority need in the SEA Region. After the declaration of TB as a Regional flagship priority, communities have increasingly contributed to the planning of priority interventions, development of training materials, and conducting community capacity-building workshops. Community members, in a first-of-its-kind engagement, are represented on key advisory committees. Community organisations took the lead in developing training modules for response to drug-resistance TB^24^ and an information brochure on TB preventive treatment (TPT) that was translated into regional languages. Community-led workshops developed a roadmap for improving coverage of TPT and for enhanced engagement during the COVID-19 pandemic. Community members also contributed to the development of the Regional Strategic Plan 2021–2025.^25^ A regional community capacity-building workshop was held in March 2023 in Nepal for meaningful engagement in the planning and monitoring of national TB programmes. All these actions have not only helped increase community participation but also incorporate community voices into TB response in the Region contributing to make it people-centred.

### Emphasis on research and innovation

Intensified research and innovation are the third pillar of the End TB Strategy. In addition to optimal implementation of existing TB control strategies, major technological breakthroughs are needed to ensure a rapid decline in TB incidence and achieve the targets by 2030. Countries in the Region have strengthened research and innovation efforts that range from clinical trials, systems review, and policy research to operational research. Some of the new tools and technologies emerging from the Region—molecular test for rapid diagnosis of TB—TrueNat, and a new antigen-based skin test for TB infection (Cy-TB test) have been endorsed by WHO.^26^^,^^27^

Thailand is among the first countries to develop a broad TB research agenda using a participatory and rigorous process involving academicians, health ministry officials and civil society representatives. Thailand is also amongst the first countries to programmatically adopt the new 6–9-month BPaL/M (bedaquiline, pretomanid, linezolid and moxifloxacin) fully oral regimen for MDR-TB.

There is ongoing research on shorter all-oral regimens for drug-resistant TB in India, Indonesia, and Myanmar.

As part of epidemiological research, prevalence surveys have been completed in India Myanmar and Nepal, and planned in Indonesia and Thailand. Drug-resistance surveys have been completed in Indonesia, Myanmar, and Timor-Leste. Vulnerability assessments to understand TB epidemiology by identifying those at risk of developing TB are underway in Timor-Leste.

A phase III clinical trial of the TB preventive vaccine for household contacts of patients along with a post-infection vaccine to prevent recurrence is ongoing in India, run by the ICMR.

The region also has several other examples of innovative approaches that harness technology to improve access to services, treatment adherence, and support for persons with TB (Box 2).

Box 2 **Innovation from the South-East Asia Region.**

1 Use of digital technologies such as digital X-rays along with computer-aided diagnosis, ‘99-DOTS’ to improve adherence, and electronic recording and reporting.
2 Use of digital technologies for direct benefit transfer to TB patients during treatment.
3 Use of social media to maintain contact with TB patients and peer support by forming social groups.
4 Innovative models of patient support such as the ‘adopt a patient’ approach in India.

## Progress towards ending TB in the Region

TB treatment coverage defined as the percentage of notified cases out of all estimated cases, had gradually reached 78% (2019) in the Region^28^ which is 25% more than the estimated coverage in 2016–pre-flagship year. There was also a 6% increase in treatment success rates till 2020.

**Fig. 2:**
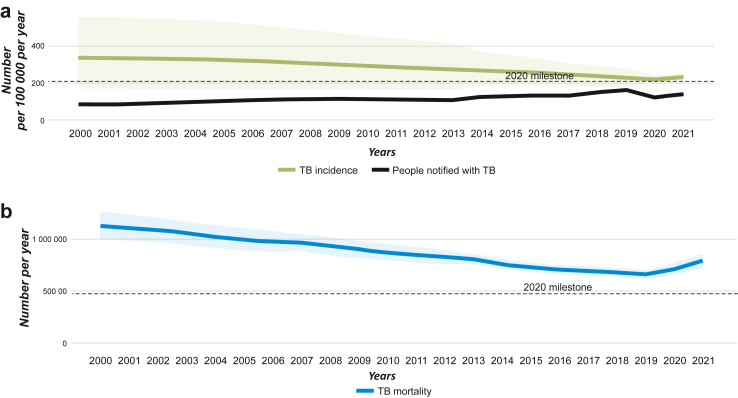
a) Trends in case notification and estimated incidence in the South-East Asia Region. b) Trends in mortality in the South-East Asia Region.

Source: WHO. Global tuberculosis report 2022. Geneva^1^ (shaded area are the uncertainty levels).

A steady albeit slow decline in the TB incidence rate of 2–3% annually had been observed in the Region till 2020 (Fig. 2a).^1^ However, only Myanmar is estimated to have reached the 2020 target for incidence rate decline. Estimated TB deaths declined from 725,000 per year in 2015 to 657,000 in 2019, which is almost a 9% decline (Fig. 2b).^1^ Bangladesh and Thailand were on track till 2020 to reach the mortality decline targets.^1^

The COVID-19 pandemic disrupted access to health services, reversing the gains made towards ending TB. During the pandemic, there was decreased detection and notification of TB patients, with a subsequent fall in those placed on treatment. Shortfalls in TB case notifications were reported in the SE Asia Region for both 2020 and 2021. In 2020, 2.6 million people with a new or relapse episode of TB were notified, down by 24% as compared to reported notifications in 2019. The decline was higher than the overall global shortfall of 18%.^29^ In 2021, there was a partial recovery, to 3.0 million, but it was still 12% less than the number of reported patients in 2019 before the pandemic. The pandemic also aggravated major TB determinants such as undernutrition and poverty.

An estimated 7 million people are likely to develop TB and 1.5 million additional deaths due to TB between 2021 and 2025 in the Region because of COVID-19-related disruptions unless urgent action is taken to cover the lost ground on missing diagnoses and notifications.^30^

Preliminary estimates available with the WHO indicate that there was a rebound in TB case notifications in several countries in 2022, reaching higher levels than in 2019 and therefore showing early signs of recovery in the post COVID-19 pandemic period. At the current pace and level of efforts, the Region is poised to miss the 2030 targets for ending TB.^31^, ^32^, ^33^

## Discussion

TB response gained the necessary momentum with the adoption of the End TB Strategy in 2014 and the SDGs in 2015. The End TB Strategy calls for an 80% reduction in TB incidence, a 90% reduction in TB deaths by 2030 and zero catastrophic costs among TB-affected persons and their families from 2020 onwards.^34^ The Strategy is based on three pillars: (i) integrated, patient-centred care and prevention; (ii) bold policies and supportive systems; and (iii) intensified research and innovation.

WHO Director General's Flagship Initiative to End TB launched in March 2023^35^ is an ambitious step for improving coverage of people-centric services. The initiative calls for reaching 90% of people with TB treatment between 2023 and 2027; 100% of people with TB tested initially with a WHO-recommended diagnostic test; 90% of eligible people provided with TB preventive treatment; 100% eligible people with TB having access to health and social benefits package; licensing of at least one new TB vaccine by 2025; and investments towards ending TB reaching US$22 billion annually by 2027. These global targets need to be adapted and aligned with SEA Regional targets and needs.

Improving coverage and realising the goals requires giving high priority to TB programmes at the highest political, policy and decision-making levels. This has been achieved in the SEA Region through a series of high-level meetings held in 2017, 2018, 2021 and more recently in 2023 with active ministerial-level participation of Member States that translated subsequently into country-level action. The commitment needs to be sustained for the coming years to ensure the desired focus on ending TB.

Social determinants play an important role in the emergence of TB disease. An analysis of TB in high burden countries identified under-nutrition, diabetes, smoking, alcohol use and HIV/AIDS as the leading population-level attributable factors for TB. The analysis found that, of all new TB patients in the SEA Region, there were close to a million attributable to undernutrition in 2021.^36^ There is a need to address these determinants through intersectoral collaborations and coordination that goes beyond the health sector.

At the implementation level, the major challenges faced in the Region relate to access and outreach, sociocultural barriers, and the requisite resources for the implementation of a comprehensive strategy in each of the countries.

**Fig. 3:**
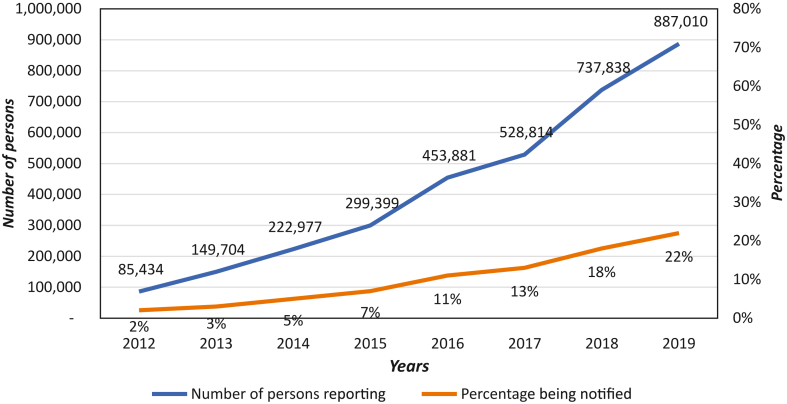
**Private provider notifications—number being treated, and percentage being notified. (Total of Bangladesh, India, Indonesia, and Myanmar)**.

Source: TB PPM learning network. Available from: https://www.tbppm.org/.^40^

TB case finding in the Region is mostly facility-based and confined to those spontaneously presenting with symptoms (passive case finding). This is inadequate because prevalence surveys in Bangladesh and Indonesia have shown that over 40% of patients with symptoms do not seek care.^37^ In the national prevalence survey in Nepal (2018–2019), over 70% of TB cases had no reported symptoms but had abnormal chest X-rays.^38^ Therefore, active outreach is needed to supplement the current passive approach of case finding. An estimated three-quarters of people in South-East Asia seek healthcare in the private and informal sector as the first point of contact,^39^ including those with TB. Although private-sector TB notifications have shown a ten-folds increase since 2012, they still represent only 22% of the estimated cases reported in the sector (Fig. 3).

Another major gap observed in the Region relates to access to services by TB patients in congregate settings–prisons, slums, hostels, and barracks is.^41^ People residing in such settings do not always have access to TB care because of a different, or absence, of appropriate health systems and services, and associated stigma and discrimination. Additionally, close contacts of TB patients in such settings are at higher risk of developing the disease as compared to the general population^42^, ^43^, ^44^ but do not always receive the necessary attention.

Stigma and discrimination associated with the disease specifically in such communities form a barrier to addressing the TB situation. Stigma has been associated with delayed care-seeking by patients and non-adherence to treatment.^45^, ^46^, ^47^ However, the issue has received variable attention in the countries mainly because of the lack of organised affected communities and survivor groups in many countries.

The earmarked budgetary allocations have only meant an increase in fiscal space or the “potential” of national TB programmes to receive funding as per the planned activities for the year. The actual allocations or the funding received by national TB programmes in the region for 2021 was only US$ 635 million. This was 50% of the estimated budget for that year. The budget allocations have been skewed towards biomedical approaches. For the past 3 years (specify the period), around 65% of the budget has been allocated for diagnostic and treatment needs,^48^ leaving little space for other system interventions that address the determinants and comorbidities. Such distortions can potentially impact the provision of social protection mechanisms to mitigate catastrophic costs for TB patients and their families. According to the Regional Strategic Plan towards ending TB, 2021–2025,^25^ an estimated additional US$ 1.6 billion is required annually to meet the regional targets for ending TB. With the current funding landscape, the additional funding required will be difficult, if not impossible, to mobilize because of competing priorities for domestic resources and international funding not enough to match the gaps. We need to look at opportunities for efficiencies through integrated approaches that address multiple diseases.^49^

## The way forward and conclusions

Efforts to end TB in the SEA Region requires renewed efforts, in the wake of setback suffered during the COVID-19 pandemic. Accelerating progress towards ending TB would need more than a biomedical approach, which has traditionally focused on strengthening the diagnostic and treatment machinery and capacity building of health-care professionals. Country strategies need to be inclusive of addressing underlying comorbidities and social determinants of TB like undernutrition, as shown in the RATIONS 9 Reducing Activation of Tuberculosis by Improvement of Nutritional Status trial.^50^ The trial shows that improved nutrition among contacts of TB patients can reduce the incidence by nearly half. The trial also establish links between early weight gain in underweight TB patients with improved treatment outcomes and reduced mortality.^50^

Therefore, ending TB requires a multisectoral concerted response. Member States of the Region need to consider the formation of high-level coordination mechanisms or commissions for inter-ministerial and inter-sectoral coordination to ensure accountability for actions. The countries in the Region have endorsed the South-East Asia Regional Strategic Plan, which is aligned with the global 2030 targets towards ending TB. The document sets out the roles for the health and non-health sectors and moves beyond the biomedical approach towards ending TB. The priorities set out are divided into three layers of action:

1. *Efforts to be undertaken within TB programmes:* These include the specific approaches required to improve people-centred access to services incorporating modern tools and technologies. Programmes need to pursue active case finding in populations at high risk of TB (e.g., contacts) to complement passive case finding. The case-finding activities should use highly sensitive tools such as Chest X-ray for screening and molecular diagnostics for confirmation and drug-susceptibility testing, followed by initiating all patients on appropriate treatment using newer drugs and regimens.
2. *Those to be undertaken beyond TB programmes but within the health sector:* Working within integrated primary health services that address the determinants of health as well as comorbidities such as diabetes, HIV and tobacco consumption; and empowering individuals, families and communities to be in the driver's seat for their health.^51^ This also requires expansion towards non-state sector entities providing health care and prevention services, through comprehensive Universal Health Coverage frameworks.
3. *Those that need to be undertaken beyond the health sector:* Collaboration is required among ministries of labour, social protection, family and welfare to ensure people with TB can access existing social protection mechanisms; the ministry of education for sharing accurate knowledge; with the ministry of external affairs to address cross-border collaboration; with the ministry of home/internal security to reach out to migrants; with the ministry of justice to approach prisoners; with the administration services of large metropolitan areas to address needs of people in slums with special programmes of housing, nutrition, preventive and curative accessible services, and likewise for other sectors. As better nutrition is crucially important for the Region, there is a need to address it on two fronts: among TB patients and their households by providing food or cash support for food, that would facilitate treatment adherence and prevention of TB among household contacts; and, in a broader perspective, mapping and identifying communities suffering from undernourishment to provide nutritional support and prevent TB and other undernutrition-related conditions. This also means the provision of social protection and programmes to eliminate food insecurity.

All national and international agencies must come together to capitalise on the Regional Flagship and partner for synergies towards ending TB in the highest TB burden Region. To achieve the acceleration towards ending TB in the Region, there is a need to intensify efforts to prevent, detect and treat TB using strategies that support patients and their families. It is crucially important that the TB response continues as a Regional Flagship Priority in the immediate and long-term future to end TB in the region.

## Contributors

All authors contributed to the development of this manuscript which is based on the Regional strategic plan towards ending TB in the WHO South-East Asia Region: 2021–2025 (RSP). VB led the development of the first draft under the guidance of MR and SR, who conceptualised the manuscript. MS led the process of development of RSP during her work in SEARO and provided inputs including edits during NA and SM developed the mathematical models used in the manuscript. All authors contributed technically based on expertise and experience in respective field areas including inputs from literature review during the development of RSP as well as reviewed the manuscript.

## Declaration of interests

VB, SR, MS, MKR and AT are WHO staff, while NA and SM were consultants for the modelling exercise. All other authors are members of the Southeast Asia Regional Strategic and Technical Advisory Group, and contributed in their advisory role without any associated honorarium. The authors declare no competing interest in production of this manuscript.
